# GPX4 Alleviates Diabetes Mellitus-Induced Erectile Dysfunction by Inhibiting Ferroptosis

**DOI:** 10.3390/antiox11101896

**Published:** 2022-09-25

**Authors:** Wenchao Xu, Taotao Sun, Jiaxin Wang, Tao Wang, Shaogang Wang, Jihong Liu, Hao Li

**Affiliations:** 1 Department of Urology, Tongji Hospital, Tongji Medical College, Huazhong University of Science and Technology, Wuhan 430030, China; 2 Institute of Urology, Tongji Hospital, Tongji Medical College, Huazhong University of Science and Technology, Wuhan 430030, China

**Keywords:** GPX4, ferroptosis, diabetes mellitus, erectile dysfunction

## Abstract

Pharmacological therapy of diabetes mellitus-induced erectile dysfunction (DMED) is intractable owig to the poor response to phosphodiesterase type 5 inhibitors (PDE5i). The surge in the number of diabetic patients makes it extremely urgent to find a novel therapy for DMED. Ferroptosis is a recently discovered form of cell death evoked by lipid peroxidation and is related to several diabetic complications. GPX4, an important phospholipid hydroperoxidase, can alleviate ferroptosis and maintain redox balance via reducing lipid peroxides. However, whether GPX4 can be a prospective target of DMED needs to be determined. Fifty rats were randomly divided into control group, DMED group, DMED + negative control group (DMED + NC group), DMED + low-dose group (1 × 10^6^ infectious units), and DMED + high-dose group (2 × 10^6^ infectious units). Erectile function was assessed 4 weeks after intracavernous injection of GPX4 or negative control lentivirus. The penile shafts were collected for subsequent molecular biological and histological analysis. The results demonstrated that erectile function of the rats in DMED and DMED + NC groups was extremely impaired and was improved in a dose-dependent manner with GPX4 lentivirus (GPX4-LV) injection. Additionally, upregulation of the ACSL4-LPCAT3-LOX pathway, iron overload, oxidative stress, fibrosis, and decreased endothelial and smooth muscle cell numbers were observed in the corpus cavernosum of DMED group. Meanwhile, the nitric oxide (NO)/cyclic guanosine monophosphate (cGMP) pathway was inhibited, and the Ras homolog gene family member A (RhoA)/Rho-associated protein kinase (ROCK) pathway was promoted in DMED rats. The above histologic alterations and related molecular changes were alleviated after GPX4-LV injection. The results revealed that GPX4 improved erectile function by modulating ferroptosis during DMED progression. This finding is of paramount significance in deciphering the molecular mechanism of hyperglycemia-induced ferroptosis, thereby providing a prospective target for preventing the development of DMED.

## 1. Introduction

Erectile dysfunction (ED), a prevalent andrological disorder, afflicts the diabetic population by up to 75% [1]. According to the International Diabetes Federation, the number of diabetes individuals worldwide is anticipated to expand by 25% in 2030 [2]. In addition to its high prevalence, ED manifests itself earlier and with greater severity in diabetic patients than in the general population [3]. This phenomenon conveys that the treatment and management of diabetes mellitus-induced erectile dysfunction (DMED) have emerged as an inevitable topic. Currently, phosphodiesterase type 5 inhibitor (PDE5i) is used as the first-line therapy for ED. Despite the fact that most ED patients display a significant improvement in erectile function while using PDE5i, DMED patients only experience a 44% response rate [4]. Therefore, discovering innovative and efficient therapeutic targets for DMED is a pressing issue in the field of uro-andrology.

A growing body of evidence suggests that iron imbalance is the common denominator in many types of diabetic complications [5,6]. Iron and iron-containing proteins are involved in many biological processes. As others have reported, diabetic wound healing is intently grappling with iron deficiency. Iron supplementation can improve the wound healing of patients who suffer from iron deficiency [5]. Conversely, iron overload can also cause ferroptosis [7] and diabetic complications via oxidative damage, but the exact mechanisms are not clear [8]. In the past ten years, ferroptosis, an iron-dependent and lipid peroxidation-induced cell death, has drawn increasing attention [9,10]. Its main feature is the metabolic abnormality of intracellular lipid oxidation, which generates a significant number of lipid peroxides under the catalysis of iron, violates the redox balance, and ultimately triggers cell death [11]. For example, knocking down transferrin receptor, which import iron from the extracellular setting, alleviates diabetic kidney disease and insulin resistance [12,13] and decreases sensitivity to ferroptosis. Additionally, polyunsaturated fatty acids (PUFA) are susceptible to lipid peroxidation and cells supplemented with PUFA are sensitized to ferroptosis. Thus, iron metabolism and lipid metabolism impact the execution of ferroptosis [9].

As a form of programmed cell death, ferroptosis contributes to the maintenance of normal physiological functions. Dysregulation of ferroptosis, however, has been linked to various diseases, including diabetic complications [14], neuroinflammation [15], and ischemia-reperfusion injury [16]. Luo et al. [17] reported that high glucose induced ferroptosis in vascular endothelial cells, resulting in increased lipid reactive oxygen species (ROS) and decreased cell viability. In addition, Ma, et al. [18] found that pretreatment with metformin attenuates palmitic acid-stimulated ferroptosis concomitant with increased calcium deposition in smooth muscle cells. Considering these facts, endothelial cells and smooth muscle cells, the main cell types in corpus cavernosum involved in penile erection, presumably undergo ferroptosis under the influence of diabetes, hence contributing to the development of DMED.

Lipid peroxides are enforcers of ferroptosis, and their removal mainly depends on glutathione peroxidase 4 (GPX4). GPX4 is a terminal molecule of the glutathione metabolic pathway, which is the earliest discovered and the most studied ferroptosis pathway [19,20]. The cystine-glutamate anti-transporter (system X_c_^-^) is composed of transmembrane proteins SLC7A11 and SLC3A2, which can exchange intracellular glutamate and extracellular cystine [21]. After cystine enters cells, thioredoxin or glutathione reduce cystine to cysteine, which is subsequently involved in the synthesis of glutathione (GSH) [22,23]. GSH is an important antioxidant that reduces lipid peroxides to maintain redox stability under the catalysis of GPX4. Studies have shown that GPX4 is an indispensable enzyme that inhibits ferroptosis. Reduction in GPX4 is the main cause of ferroptosis and is also regarded as a marker of ferroptosis [24].

Therefore, we speculate that ferroptosis may be a driving factor in the development of DMED and that upregulation of GPX4 could reduce ferroptosis and improve the function of cellular components such as cavernous endothelial cells and smooth muscle cells, thereby enhancing the erection function. In this study, the rat model of DMED was established and GPX4 lentivirus was injected to verify the role of ferroptosis in DMED and to explore its mechanism.

## 2. Materials and Methods

### 2.1. Lentivirus Construction, Package, and Examination

The sequence of rat GPX4 was obtained from NCBI reference genome (NM_017165, https://www.ncbi.nlm.nih.gov/gene/?term=NM_017165, accessed on 1 August 2021). The lentiviral vectors used in this study are GV707, pHelper 1.0, and pHelper 2.0. The vector information of GV707 can be found on the GeneChem website (http://www.genechem.com.cn, accessed on 1 August 2021). Human embryonic kidney 293T (HEK-293T) cells were placed in 10 cm plates, and cells with a confluence of 70–80% were used for transfection. HEK-293T cells were cultured in serum-free RPMI1640 medium two hours before transfection. Specifically, the plasmid DNA (20 μg for GV707, 15 μg for pHelper 1.0, and 10 μg for pHelper 2.0) and transfection reagents from GeneChem Company (Shanghai, China) were diluted in serum-free RPMI1640 medium to a total volume of 1 mL, followed by 15 min incubation at room temperature. Plasmid-transfection reagent mix was then added into HEK-293T cells with incubation for 6–8 h at 37 °C, which were then seeded with complete medium. Cell suspension underwent centrifugation (4000× *g*) for 10 min in 4 °C. Supernatants abundant in lentivirus were collected and then filtered by 0.45 μm syringe filter.

Lentivirus was then condensed, harvested, and restored at −80 °C; this was then injected intraperitoneally into DMED rats. The lentivirus without the transgene was used as a negative control and was produced in the same manner. Nest polymerase chain reaction, limulus amebocyte lysate chromogenic assay, and HEK-293T culture were used to examine contamination. A titer assay was performed as previously reported, with modifications [25]. HEK-293T cells were seeded in 96-well plates (4 × 10^4^ cells/well in 100 μL) and cultured for 24 h. The medium (90 μL) was removed. The original virus solution (10 μL) and 90 μL serum-free medium were mixed, and 90 μL consecutive diluted virus solutions at dilutions of 100, 10^−1^, 10^−2^, 10^−3^, 10^−4^, 10^−5^, and 10^−6^ were added to the cells. After 72 h, puromycin (5 μg/mL) was added for another 24 h, and the viral titer was determined by cell counting. Viral titer was calculated with the following formula: Titer (units/mL) = 1000 × alive cells/original virus solution (μL). All the above lentiviruses were designed and synthesized by GeneChem Company.

### 2.2. Animal and Experimental Designs

Fifty eight-week-old male Sprague-Dawley (SD) rats were provided by the Experimental Animal Center of Tongji Hospital, Tongji Medical College, Huazhong University of Science and Technology. This study was approved by the Institutional Research Ethics Committee at Tongji Hospital, Tongji Medical College, Huazhong University of Science and Technology (TJH-202106003). As previously reported, the rats with DMED were modeled and identified [26]. Briefly, forty-two rats received a single intraperitoneal injection of 1% streptozotocin solution (Sigma-Aldrich, S0130, St. Louis, MO, USA) (65 mg/kg), while citrate buffer solution (0.1 mol/L, pH 4.5) was injected intraperitoneally into the remaining eight rats. Fasting blood glucose levels were examined 72 h following the injection. Those rats with fasting blood glucose levels exceeding 16.7 mmol/L were identified as diabetic. Eight weeks after the DM model was established, 39 diabetic rats survived. Subsequently, an apomorphine test was undertaken to identify rats with DMED, and rats with negative results were treated as DMED, which preliminarily represented successful modeling [27,28]. Those with ED (*n* = 32) according to the test were further divided into DMED group, DMED + negative control group (DMED + NC group), DMED + low-dose group (DMED + Low group), and DMED + high-dose group (DMED + High group) randomly, with eight rats in each group. Rats in DMED + Low and DMED + High groups were treated with GPX4 lentivirus by intracavernous injection (1 × 10^6^ or 2 × 10^6^ infectious units) [29], while the DMED + NC group was injected with the equivalent amount of negative control virus. Both the initial and final weight, as well as the blood glucose level, were documented.

### 2.3. Erectile Function Evaluation

Erectile function was assessed by the max intracavernous pressure (ICP)/mean arterial pressure (MAP) ratio and the total ICP calculated by area under the curve. All rats received an intraperitoneal injection of sodium pentobarbital (30 mg/kg). The cavernous nerve was painstakingly exposed, and the corpora cavernosa was meticulously isolated. To assess ICP, a 25 G needle with 100 U/mL heparin solution was slowly inserted into the proximal corpus cavernosum. Carotid artery was catheterized with a PE-10 tube to record the arterial pressure. A biosignal acquisition and processing system (BL-420F, Techman Soft, Chengdu, China) coupled the two devices together. ICP and MAP were monitored while stimulating the cavernous nerve with bipolar stainless-steel electrodes. An ICP below 60 mmHg and a max ICP/MAP ratio less than 0.5 are deemed as a successful DMED model. All rats had their penile shafts collected for future study.

### 2.4. Histologic Assessment

#### 2.4.1. Fluorescence Staining

For immunofluorescence, resected penile tissues were fixed in 4% paraformaldehyde overnight at 4 °C, then treated with gradient sucrose and embedded in O.C.T. (Sakura Finetek, Tokyo, Japan). Tissue sections (5 μm) were cut and subjected to antigen retrieval with 0.01 M sodium citrate buffer in a microwave oven. After blocking, sections were incubated overnight at 4 °C with primary antibodies (Table S1). Then, slides were washed and incubated with secondary antibodies.

ROS in the corpus cavernosum was detected using a fluorescent probe (Dihydroethidium, DHE). The tissue sections were incubated with DHE probe for 30 min at room temperature. Subsequently, DAPI was used to stain the cell nuclei. Then, slides were observed under a fluorescence microscope.

#### 2.4.2. Immunohistochemistry and Masson’s Trichrome Staining

The penile tissues of rat were dissected and fixed overnight at 4 °C, and then stored in 70% ethanol. Samples were dehydrated for paraffin embedding. Five-micrometer sections were stained after dewaxing and rehydration. Immunohistochemistry staining of penile tissue sections was performed using corresponding primary antibodies (Table S1). The slides were subsequently incubated with a biotinylated secondary antibody. To evaluate changes in tissue structure, Masson’s trichrome staining was performed based on the manufacturer’s recommendations. Additionally, the ratio of smooth muscle to collagen in the corpus cavernosum was quantified.

#### 2.4.3. Prussian Blue Staining

Prussian blue staining was utilized to assess the distribution of Fe^3+^. Briefly, 5 μm penile sections were deparaffinized, dehydrated, and exposed to a 1:1 (*v/v*) mixture of potassium ferrocyanide (2%) and hydrochloric acid (2%) for 20 min. The sections were then rinsed with water for 2 min and fastened with Fast Red for 5 min at room temperature. Finally, the sections were washed sequentially with water and treated with alcohol and xylene.

#### 2.4.4. Transmission Electron Microscopy

Samples of corpus cavernosum (1 mm × 2 mm × 2 mm) were swiftly resected and instantaneously fixed in 3% phosphate-glutaraldehyde. We then postfixed, embedded, cut, and mounted the samples, which were then observed using a Talos L120C transmission electron microscope (Thermo Fisher, Waltham, MA, USA).

### 2.5. Western Blotting

As previously published, protein expression levels were determined by western blotting [26,30]. We used corresponding primary antibodies (Table S1) to detect protein expression at 4 °C overnight. After washing the membranes, corresponding second antibodies were used for 1 h at room temperature. Finally, the protein bands were developed with Western ECL Substrate. Densitometry value of bands was analyzed with ImageJ 1.53 (National Institute of Health, Bethesda, MD, USA).

### 2.6. Oxidative Stress Levels and Iron Contents Detection

Following the manufacturer’s instructions, oxidative stress levels in corpus cavernosum were assessed by malondialdehyde (MDA), superoxide dismutase (SOD), glutathione (GSH/GSSG), and glutathione peroxidase (GSH-Px) levels (Nanjing Jiancheng, Nanjing, China). Total protein concentrations were used to normalize oxidative stress levels. The iron content of corpus cavernosum was measured according to manufacturer’s recommendation (Nanjing Jiancheng, Nanjing, China).

### 2.7. Nitric Oxide (NO) and Cyclic Guanosine Monophosphate (cGMP) Levels

Penile supernatants were gathered after tissue homogenizing in lysis buffer for NO and cGMP detection. The total NO content was evaluated using a nitrate-nitrite assay kit (Nanjing Jiancheng, China) according to the standard protocol. An ELISA kit (Nanjing Jiancheng, China) was applied to measure cGMP levels as described. Both NO and cGMP levels were normalized to the protein levels.

### 2.8. Statistical Analysis

The results are presented as the mean ± standard deviation. One-way ANOVA followed by Tukey’s post hoc test was used to compare multiple groups. Linear regression was used to investigate the relationship between GPX4 expression level and erectile function parameters. All statistical analyses were conducted using GraphPad Prism 8.3.0, and statistically significance was considered as *p* < 0.05.

## 3. Results

### 3.1. GPX4-LV Injection Had No Effect on Metabolic Indexes

**Figure 1:**
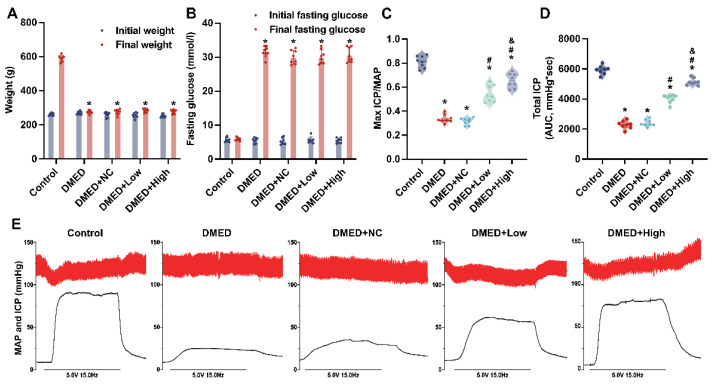
Assessment of metabolic indexes and erectile function in rats. (**A**) Initial and final weights for each group; (**B**) Initial and final fasting glucose levels for each group; (**C**) Total ICP and (**D**) the ratio of max ICP/MAP for each group; (**E**) Representative recordings of ICP and MAP during 1 min electrical stimulation at 5.0 V. * *p* < 0.05 compared with the control group. ^#^ *p* < 0.05 compared with the DMED group. ^&^ *p* < 0.05 compared with the DMED + low group.

Compared to the control group, the weight of DMED rats dropped dramatically, while the blood glucose levels of DMED rats rose significantly at the 8th week after modeling (Figure 1A,B). The weight and glucose levels of rats were measured after 4 weeks of treatment with GPX4-LV injection. As shown, there was no obvious difference in these parameters among the four groups with DM, which showed that GPX4-LV injection did not affect the weight or glucose levels of rats.

### 3.2. GPX4-LV Injection Improved Erectile Function in a Dose-Dependent Manner

ICP/MAP less than 0.5 and ICP below 60 mmHg were achieved in the DMED rat model (Figure 1C–E). The erectile function in the DMED group (Max ICP/MAP 0.337 ± 0.034; total ICP 2289 ± 258.9) was significantly lower than that in control group (Max ICP/MAP 0.812 ± 0.049; total ICP 5924 ± 284.0), and these ratios improved after GPX4-LV injection. Specifically, the max ICP/MAP (0.658 ± 0.060) and the total ICP (5141 ± 234.0) of DMED + high group showed a more obvious improvement than the DMED + low group (Max ICP/MAP 0.545 ± 0.069; total ICP 4027 ± 277.5). The data indicate that DMED model was successfully established and that GPX4-LV injection could partially prevent erectile dysfunction in DMED rats in a dose-dependent manner.

### 3.3. GPX4-LV Injection Alleviated Ferroptosis in the Corpus Cavernosum

**Figure 2:**
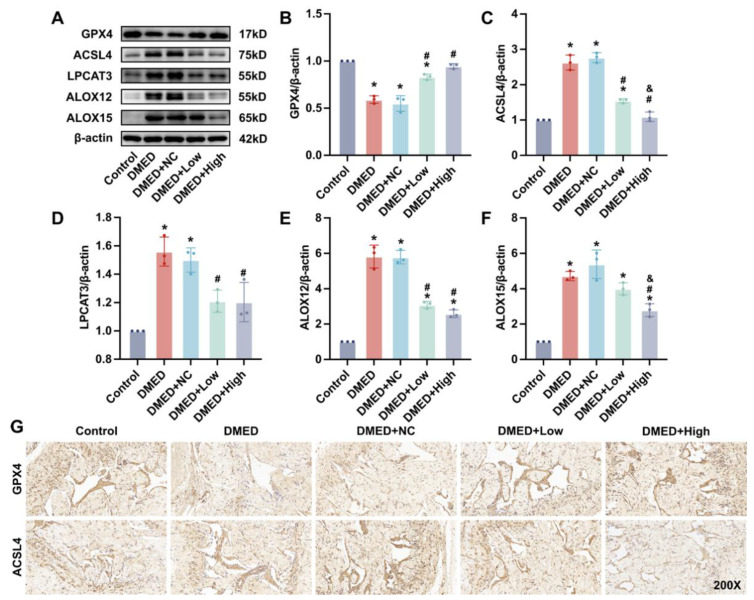
Effect of GPX4-LV injection on ferroptosis in diabetes mellitus-induced erectile dysfunction (DMED) rats. Representative immunoblot (**A**) and semi-quantification (**B**–**F**) of GPX4, ACSL4, LPCAT3, ALOX12, and ALOX15 in the corpus cavernosum after 4 weeks of treatment; (**G**) Representative immunohistochemistry (200×) of GPX4 and ACSL4 in corpus cavernosum. * *p* < 0.05 compared with the control group. ^#^ *p* < 0.05 compared with the DMED group. ^&^ *p* < 0.05 compared with the DMED + low group.

GPX4 is a keystone of ferroptosis, so we next investigated whether ferroptosis exists in the corpus cavernosum of DMED rats and whether GPX4 could improve erectile function by inhibiting ferroptosis. Western blotting results confirmed that the protein expression of GPX4 was significantly reduced in the DMED and DMED + NC groups, suggesting that ferroptosis is involved in DMED (Figure 2A,B). After injecting GPX4 lentivirus, GPX4 content was obviously elevated, confirming that our injection was successful. Moreover, we hypothesized that the ACSL4-LPCAT3-LOX pathway, which accelerates ferroptosis, would be influenced by GPX4-LV injection. Consistent with our expectation, the ACSL4-LPCAT3-LOX pathway was upregulated in the DMED group, while GPX4-LV injection decreased its protein expression (Figure 2A–F). Additionally, immunohistochemistry staining was performed to confirm the above findings. As shown in Figure 2G, GPX4 lentivirus restored downregulated GPX4 expression caused by hyperglycemia in corpus cavernosum. Conversely, increased expression of ACSL4 was inhibited by GPX4 lentivirus injection. Therefore, GPX4-LV injection alleviated ferroptosis by increasing GPX4 and decreasing ferroptosis-promoting proteins in the corpus cavernosum.

### 3.4. GPX4-LV Injection Ameliorated Iron Overload and Oxidative Stress

**Figure 3:**
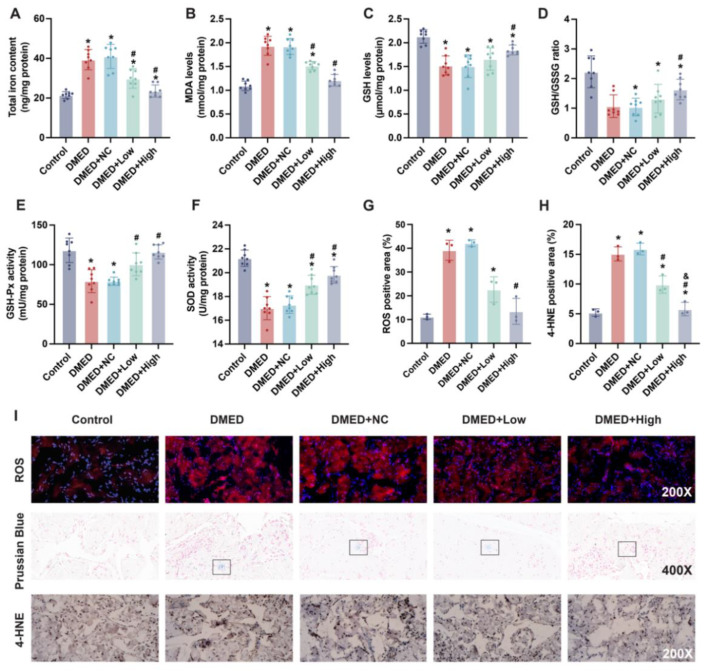
Effect of GPX4-LV injection on iron overload and oxidative stress in DMED rats. (**A**) Total iron content, (**B**) MDA levels, (**C**) GSH levels, (**D**) GSH/GSSG ratio, (**E**) GSH-Px activity, and (**F**) SOD activity in all five groups. Semi-quantification (**G**,**H**) and representative results (**I**) of ROS (200×), Prussian Blue (400×) and 4-HNE (200×) staining. * *p* < 0.05 compared with the control group. ^#^ *p* < 0.05 compared with the DMED group. ^&^ *p* < 0.05 compared with the DMED + low group.

Iron also plays an important role in ferroptosis, so the content of iron was measured. Consistent with the hypothesis that ferroptosis contributes to DMED, the total iron content was increased in the DMED group, while it was significantly decreased with GPX4-LV injection (Figure 3A). Moreover, Prussian blue staining was used to detect iron deposits. As shown in Figure 3I, some staining foci could be seen in the DMED and DMED + NC groups. However, high-dose GPX4-LV injection apparently mitigated this situation. Lipid peroxidation is another essential process for conducting ferroptosis, in which MDA and 4-HNE could partially represent the level of lipid peroxidation. DMED and DMED + NC groups exhibited higher MDA and 4-HNE levels than the control group, which partly decreased with GPX4-LV injection (Figure 3B,H,I). ROS are downstream of lipid peroxidation, which can lead to ferroptosis. To further illuminate the mechanisms of ferroptosis, DHE staining was used to determine the level of ROS. The ROS-positive area was increased in the DMED and DMED + NC groups, while GPX4-LV injection decreased it significantly (Figure 3G,I). It has been reported that some antioxidative species, such as GSH, GSH-Px, and SOD, can scavenge ROS. Therefore, we hypothesized that low levels of antioxidative species in hyperglycemia could not efficiently eliminate the negative effect of ROS on ferroptosis in smooth muscle cells and endothelial cells. These results show that GSH levels, GSH/GSSG ratio, GSH-Px activity, and SOD activity were lowered in the DMED and DMED + NC groups, but were reversed by GPX4-LV injection (Figure 3C–F).

### 3.5. GPX4-LV Injection Restored Endothelial and Smooth Muscle Cell Numbers in the Corpus Cavernosum

**Figure 4:**
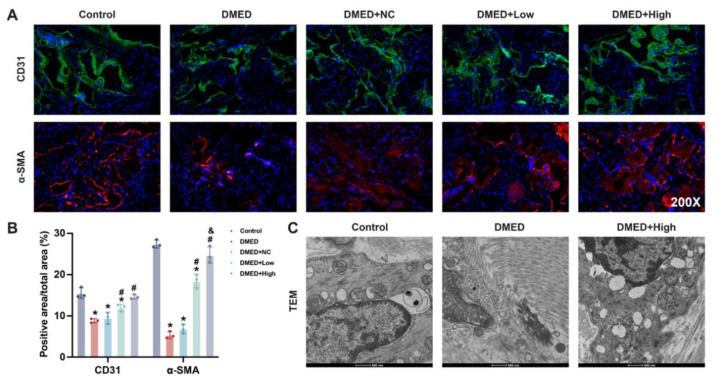
Effect of GPX4-LV injection on endothelial and smooth muscle content in DMED rats. Representative results of immunofluorescence for α-SMA (**A**) and CD31 (**B**) (200×). (**C**) Representative results of transmission electron microscopy in the three groups. * *p* < 0.05 compared with the control group. ^#^ *p* < 0.05 compared with the DMED group. ^&^ *p* < 0.05 compared with the DMED + low group.

To determine which types of cells respond to ferroptosis, α-SMA and CD31 (smooth muscle cell and endothelial cell markers, respectively) were stained. Immunofluorescence results demonstrate that the number of both endothelial cells and smooth muscle cells were decreased under the circumstance of hyperglycemia, whereas GPX4-LV injection protected their survival in diabetic rats (Figure 4A,B). Moreover, transmission electron microscopy was used to determine the structural change in corpus cavernosum. Under transmission electron microscope, the membranes of endothelial cells were intact, the structure was clear, the arrangement was regular, and the mitochondria were normal in control group. However, in the DMED group, the endothelial cell membrane was ruptured and swollen, and organelles were significantly reduced. We also observed mitochondria shrinking and nuclear chromatin edges gathering. After GPX4-LV injection, mitochondria were enlarged or appeared normal (Figure 4C).

### 3.6. GPX4-LV Injection Improved the NO-cGMP and RhoA-ROCK1/ROCK2 Signaling Pathway

**Figure 5:**
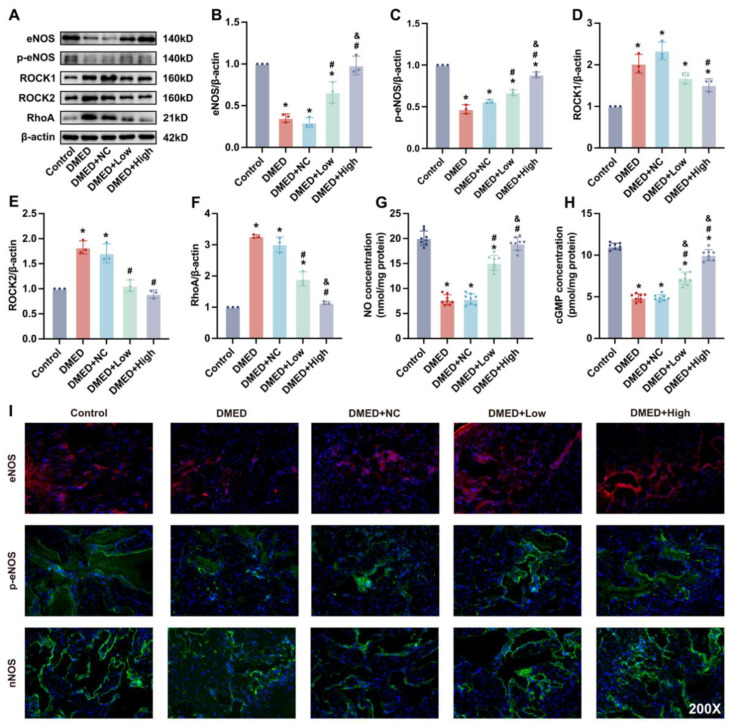
Effect of GPX4-LV injection on endothelial and smooth muscle content in DMED rats. Representative immunoblot (**A**) and semi-quantification (**B**–**F**) of eNOS, *p*-eNOS, ROCK1, RCOK2, and RhoA in corpus cavernosum; (**G**) NO production and (**H**) cGMP production in corpus cavernosum; (**I**) Representative results of immunofluorescence for eNOS, *p*-eNOS, and nNOS (200×). * *p* < 0.05 compared with the control group. ^#^ *p* < 0.05 compared with the DMED group. ^&^ *p* < 0.05 compared with the DMED + low group.

As shown above, we wondered whether endothelial and smooth muscle cell loss might influence endothelial and smooth muscle function in the corpus cavernosum of DMED rats. The NO-cGMP pathway is a major participator in endothelial function. To better understand the process, eNOS and phospho-eNOS (Serine 1177) (*p*-eNOS) were detected by Western blotting and immunofluorescence. As shown in Figure 5A–C, the results of Western blotting analysis showed that levels of eNOS and *p*-eNOS in the control group were higher than those in the other four groups. Although the levels of eNOS and *p*-eNOS after GPX4-LV injection were lower than those in the control group, they were significantly higher than those in the other DMED groups (Figure 5A–C). Consistent with the Western blotting results, the immunofluorescence results of eNOS and *p*-eNOS also showed that expression of eNOS and *p*-eNOS was decreased in the DMED group, and partially increased after GPX4-LV injection (Figure 5I). Similar results were also detected for nNOS, another important factor in the production of NO in the corpus cavernosum (Figure 5I). Moreover, NO and cGMP levels were also measured. Compared with the other groups, NO and cGMP concentrations in the DMED group were significantly decreased, and partially increased after GPX4-LV injection (Figure 5G,H).

Smooth muscle relaxation is under the control of the RhoA-ROCK1/ROCK2 signaling pathway. RhoA content was increased in the DMED and DMED + NC groups compared with the control group, but was obviously reduced after GPX4-LV injection (Figure 5A,D–F). Therefore, smooth muscle function was impaired in diabetic rats, while GPX4-LV injection partially improved smooth muscle function (Figure 5A,D–F). The above results all indicate that GPX4 can improve the NO-cGMP pathway and inhibit the RhoA-ROCK1/ROCK2 pathway in the corpus cavernosum of DMED rats.

### 3.7. GPX4-LV Injection Inhibited Fibrosis in the Corpus Cavernosum

**Figure 6:**
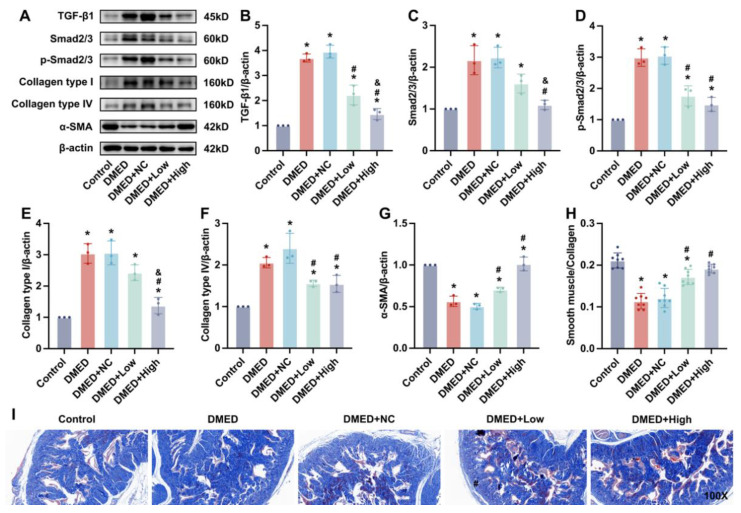
Effect of GPX4-LV injection on fibrosis in DMED rats. Representative immunoblot (**A**) and semi-quantification (**B**–**G**) of TGF-β1, Smad2/3, *p*-Smad2/3, Collagen type I and IV, and α-SMA in corpus cavernosum. Semi-quantification (**H**) and representative images of Masson’s trichrome staining (100×) (**I**). * *p* < 0.05 compared with the control group. ^#^ *p* < 0.05 compared with the DMED group. ^&^ *p* < 0.05 compared with the DMED + low group.

The expression levels of fibrosis-related factors, including TGF-β1, Smad2/3, *p*-Smad2/3, collagen I, collagen IV, and α-SMA, in penile tissue were detected (Figure 6). The content of α-SMA was decreased in the DMED and DMED + NC groups compared with the control group, while the expression was obviously increased after GPX4-LV injection (Figure 6A,G). However, the expression of the other proteins was significantly increased in the DMED and DMED + NC groups and slightly ameliorated in the DMED + low and DMED + high groups (Figure 6A–F). Furthermore, the ratio of smooth muscle to collagen was assessed by Masson’s trichrome staining. The results revealed a lower content of smooth muscle and more collagens in the DMED group than in the control group, which indicated that a worse degree of cavernous fibrosis occurred in diabetic rats, but this could be alleviated after injection of GPX4-LV (Figure 6H,I).

## 4. Discussion

**Figure 7:**
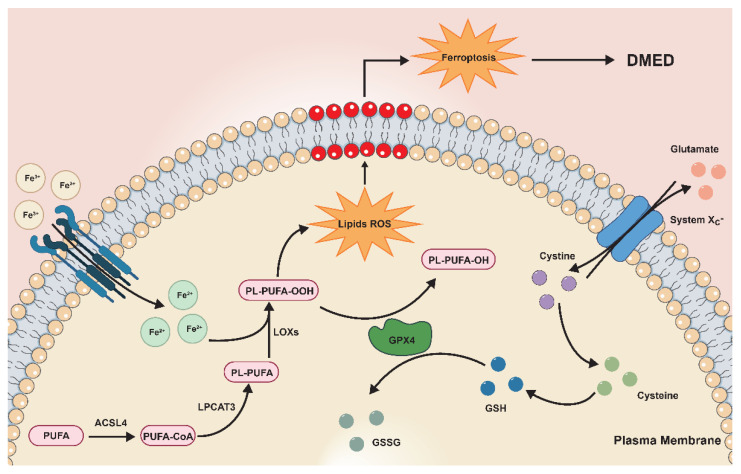
Proposed mechanism of ferroptosis in DMED. More polyunsaturated fatty acids (PUFAs) are catalyzed into lipid peroxides with the help of the upregulated ACSL4-LPCAT3-LOX pathway in DMED. The cystine-glutamate anti-transporter (system Xc-) transports cystine into the cell, which participates in the synthesis of glutathione. Less glutathione reduces lipid peroxides under the catalysis of decreased GPX4 expression in DMED. The above molecular changes were alleviated after GPX4-LV injection. Subsequently, the level of ferroptosis is reactively improved. The end result of these processes is the amelioration of erectile function in DMED.

Diabetes is a major risk factor for ED. GPX4 lentivirus was injected to explore the role of GPX4 in the development of DMED. Our work found that GPX4 was decreased in the penile tissue of diabetic rats and that GPX4-LV injection could ameliorate DMED. Further analysis showed that GPX4 expression level was positively related with erectile function (Figure S1). Ferroptosis, oxidative stress, and fibrosis were alleviated in the corpus cavernosum following GPX4-LV injection (Figure 7).

Glutathione peroxidases (GPXs) are evolutionarily highly conserved enzymes that reduce peroxides (e.g., R-OOH) to their corresponding alcohols (R-OH) using GSH as a cofactor, thereby limiting the formation of metal-dependent toxic free radicals (e.g., R-O •) [31]. Unlike other family members, GPX4 can act as a phospholipid hydroperoxidase to reduce lipid peroxides to lipid alcohols. Thus, GPX4 activity is critical for maintaining lipid homeostasis in cells, preventing the accumulation of toxic lipid ROS and thereby blocking the occurrence of ferroptosis [31]. Ferroptosis is a new-found form of cell death characterized by iron-dependent overwhelming lipid peroxidation [32]. It has been shown that ferroptosis is involved in various pathological conditions [33,34]. We also previously found that apoptosis is increased in the penis of diabetic rats, but the use of apoptosis inhibitors can only partially reduce cell death, and the improvement of penile erectile function was also not obvious, suggesting that apoptosis is not the predominant form of cell death in corpus cavernosum [26]. From this collection of evidence, we speculate that there are other forms of cell death involved in the pathogenesis of DMED. However, the exact molecular network involved in hyperglycemia-induced ferroptosis remains understudied.

To elucidate the role of GPX4 and ferroptosis, the rat model of DMED was established. After injecting streptozotocin, the DMED group showed significantly impaired erectile function in the DMED group, which was similar to previous reports. Moreover, our data showed that hyperglycemia caused changes in ferroptosis-related markers, including GPX4, ACSL4, iron, and MDA levels, in diabetic rats. Interestingly, these aberrant parameters were attenuated by GPX4-LV injection. It has been well documented that iron overload, a keystone of ferroptosis, can be observed in many types of diabetic complications [14,35], but the exact mechanism of how iron overload happens is still obscure. Proteins associated with iron import, export, storage, and turnover impact iron content in tissues [36]. It has been reported that hyperglycemia inhibits the expression of Nrf2 in diabetic cardiomyopathy [14]. Moreover, Nrf2 could stimulate the downstream expression of ferritin, which also decreased in diabetic environment and in turn increased the labile iron levels [14]. Similar with previous reports, Nrf2 expression was decreased in DMED [37,38]; thus, the Nrf2/ferritin pathway may account for iron overload in DMED. More definitive evidence and further investigation are required for aberrant iron-related molecules in DMED. Taken together, from our research, we deem that GPX4 is of paramount importance for erectile function by inhibiting ferroptosis in DMED.

Recently, it was reported that hyperglycemia or high glucose activates oxidative stress and triggers subsequent ferroptosis in many organs and cell lines. Feng et al. [39] reported that downregulation of the HIF-1α/HO-1 pathway resulted in overproduction of ROS and caused renal tubule ferroptosis in diabetic models. Wu et al. [40] reported that HMGB1 regulates intracellular oxidative stress and ferroptosis through the Nrf2 pathway in mesangial cells in response to high glucose. These findings suggest that oxidative stress plays a crucial role in both hyperglycemia and high glucose-induced ferroptosis. Indeed, our data confirms that GPX4 decreased the levels of ROS and 4-HNE, a marker of lipid peroxidation, while it increased the levels of some antioxidant species, such as GSH and SOD. These findings are in accordance with the discovery that the activity of GPX4 is critical for maintaining lipid homeostasis, preventing the accumulation of toxic ROS, and thereby inhibiting ferroptosis.

Reduction in cell numbers and function is the major mechanism in DMED. Herein, immunofluorescence results show that CD31 and α-SMA (markers of endothelial cells and smooth muscle cells, respectively) decreased. Transmission electron microscopy results suggested that the cells showed abnormal morphology. Furthermore, endothelial function depends on the NO-cGMP pathway, while RhoA-ROCK1/ROCK2 partially represents the function of smooth muscle cells. It has been reported that excess ROS reduced *p*-eNOS levels and NO content, and enhanced the RhoA-ROCK1/ROCK2 pathway. These results were consistent with our findings. Additionally, GPX4-LV injection rescued changes of these two processes. Notably, the affected phosphorylation site of eNOS that we detected was Serine 1177 (Ser1177). According to other studies, the phosphorylation level of Threonine 495 (Thr495) was elevated under high glucose stimulation, accompanied by eNOS uncoupling and increased reactive nitrogen species (RNS). Whether these aberrant changes also exist in DMED deserves further investigation. All of the above results indicate that GPX4 could improve cell numbers and function by inhibiting oxidative stress-induced ferroptosis.

Similar to previous reports [26,30], increased collagen content and fibrosis induced by hyperglycemia could impair erectile function. The results of Masson’s trichrome staining showed that the smooth muscle/collagen ratio and the smooth muscle marker α-SMA were significantly downregulated in DMED rats, along with increased fibrosis markers TGF-β, collagen types I and IV, and Smad 2/3. GPX4-LV injection inhibited fibrosis in the corpus cavernosum, confirming that ferroptosis could influence fibrosis. However, the complex and finely concrete mechanism between ferroptosis and fibrosis is still far from being resolved.

This work demonstrates that GPX4 deficiency mediates the occurrence and development of DMED. GPX4 ameliorated DMED by reducing ferroptosis, oxidative stress, and fibrosis. However, some limitations have to be addressed in our study. Firstly, we did not investigate how the expression of GPX4 was decreased in a diabetic setting. It has been reported by other studies that hyperglycemia could reduce GPX4 expression by inactivating the Nrf2/GPX4 pathway [41] or facilitating GPX4 ubiquitination by TRIM46 [42]. Whether these factors mediate GPX4 downregulation in the corpus cavernosum of diabetic rats requires further exploration. In addition, how GPX4 ameliorated cavernous fibrosis and improved endothelial and smooth muscle function was not clear. More devoted research is needed to illustrate the role of these pathways in ferroptosis-induced changes in the corpus cavernosum. Furthermore, the above experiments were conducted in streptozotocin-induced type 1 diabetes, which impeded generalization to type 2 diabetes. Further experiments are a key step to extend our results to the general diabetic population.

## 5. Conclusions

In conclusion, the results herein reveal a novel role of GPX4 in regulating ferroptosis during DMED progression. This finding has important implications for deciphering the molecular mechanism of hyperglycemia-induced ferroptosis, thereby providing a prospective target for preventing the development of DMED in clinical practice.

## Data Availability

The data presented in this study are available on request from the corresponding author.

## Supplementary Materials

The following supporting information can be downloaded at https://www.mdpi.com/article/10.3390/antiox11101896/s1. Figure S1: The relationship between GPX4 expression and erectile function parameters; Table S1: Antibodies used in this study.
