# Supplementary material for: GPX4 Alleviates Diabetes Mellitus-Induced Erectile Dysfunction by Inhibiting Ferroptosis
Source: Antioxidants (Basel). 2022 Sep 25;11(10):1896. doi: 10.3390/antiox11101896 (PMC9598206; doi:10.3390/antiox11101896)
Supplement: Supplementary file 1 [file antioxidants-11-01896-s001.zip › Table S1.pdf]

Table S1. Antibodies used in this study.

Antibody	Manufacture (catalog number)	Source	Applications
GPX4	Boster (BM5231)	Rabbit	WB
GPX4	Affinity (DF6701)	Rabbit	IHC
ACSL4	Affinity (DF12141)	Rabbit	WB
ACSL4	Proteintech (22401-1-AP)	Rabbit	IHC
LPCAT3	Abclonal (A17604)	Rabbit	WB
ALOX12	Santa Cruz (sc-365194)	Mouse	WB
ALOX15	Abclonal (A6864)	Rabbit	WB
β-Actin	Abclonal (AC026)	Rabbit	WB
4-HNE	Abcam (ab48506)	Rabbit	IHC
CD31	Servicebio (GB12063)	Mouse	IF
α-SMA	Servicebio (GB111364)	Rabbit	IF
α-SMA	Boster (BM0002)	Mouse	WB
eNOS	Affinity (AF0096)	Rabbit	WB, IF
p-eNOS	Affinity (AF3247)	Rabbit	WB, IF
ROCK1	Proteintech (21850-1-AP)	Rabbit	WB
ROCK2	Boster (PB0428)	Rabbit	WB
RhoA	Proteintech (10749-1-AP)	Rabbit	WB
nNOS	Servicebio (GB11145)	Rabbit	IF
TGF-β1	Affinity (AF1027)	Rabbit	WB
Smad2/3	Cell Signaling (D7G7)	Rabbit	WB
p-Smad2/3	Cell Signaling (D27F4)	Rabbit	WB
Collagen type I	Santa Cruz (sc-293182)	Mouse	WB
Collagen type IV	Proteintech (55131-1-AP)	Rabbit	WB
